# Novel PEFC Application for Deuterium Isotope Separation

**DOI:** 10.3390/ma10030303

**Published:** 2017-03-16

**Authors:** Hisayoshi Matsushima, Ryota Ogawa, Shota Shibuya, Mikito Ueda

**Affiliations:** Faculty of Engineering, Hokkaido University, Kita 13 Nishi 8, Sapporo, Hokkaido 060-8628, Japan; ogahdf.1993@gmail.com (R.O.); s-shibuya@frontier.hokudai.ac.jp (S.S.); mikito@eng.hokudai.ac.jp (M.U.)

**Keywords:** hydrogen isotope separation, fuel cell, CEFC, isotope exchange reaction

## Abstract

The use of a polymer electrolyte fuel cell (PEFC) with a Nafion membrane for isotopic separation of deuterium (D) was investigated. Mass analysis at the cathode side indicated that D diffused through the membrane and participated in an isotope exchange reaction. The exchange of D with protium (H) in H_2_O was facilitated by a Pt catalyst. The anodic data showed that the separation efficiency was dependent on the D concentration in the source gas, whereby the water produced during the operation of the PEFC was more enriched in D as the D concentration of the source gas was increased.

## 1. Introduction

The heavy isotopes of hydrogen, deuterium (D) and tritium (T) play essential roles in nuclear energy production [1,2]. In current heavy-water nuclear fission reactors, D is used as a neutron-moderator. Similarly, in nuclear fusion reactors, which are expected to represent the next generation of nuclear power, the reaction of D and T is responsible for the energy-production stage.

Because D and T are not directly obtainable as pure isotopes, methods to separate them from the more common, lighter isotope, protium, are required. Many researchers have studied various isotope-separation methods, including water distillation [3], molecular sieving [4], water electrolysis [5,6,7,8] and combined electrolysis catalytic exchange [9,10]. The water electrolysis yields the most effective separation but consumes enormous amounts of electricity. Such large consumption has led to a search for other methods that are more energetically efficient. In particular, a new separation technology for tritium is urgently required at the Fukushima Daiichi Nuclear Power Plant in Japan.

We previously proposed a new hydrogen-separation system: the combined electrolysis fuel cell (CEFC) process [11]. Here, hydrogen and oxygen were produced by electrolysis and used for power generation in a fuel cell. By recycling the energy generated from the produced hydrogen, the electricity consumption of the isotope separation process was reduced. More recent work has reported D separation via the hydrogen isotope effect during the anodic reaction in polymer electrolyte fuel cells (PEFCs) [12,13,14] and alkaline membrane fuel cells [15]. We reported that the water produced by these power sources was enriched in D. This was caused entirely by the kinetic isotope effect during the hydrogen oxidation reaction (HOR) on a Pt catalyst. However, several other factors must be investigated to fully realize the potential of CEFC systems. The dependency of the separation efficiency on the isotope concentration is important from the practical viewpoint. The mass balance of the isotopes in fuel cells must be strictly controlled when radioactive species are involved. Therefore, this paper focuses on measuring D separation by a PEFC and investigates the factors influencing separation at both the cathode and anode, using isotopically mixed gases with several D concentrations.

## 2. Experimental

A JARI standard cell (FC Development Corp., Tsukuba, Japan) was employed as a PEFC. The membrane electrode assembly (50 × 50 mm) was composed of Nafion electrolyte (NRE-212) and two catalytic layers loaded with platinum catalyst (Pt, 0.52 mg·cm^−2^). The PEFC was operated at 298 K and the power generation was controlled under constant current mode (0.0–1.2 A) adjusted by a variable resistor unit (PLZ 164WA, Kikusui Electronics Corp., Yokohama, Japan).

**Figure 1:**
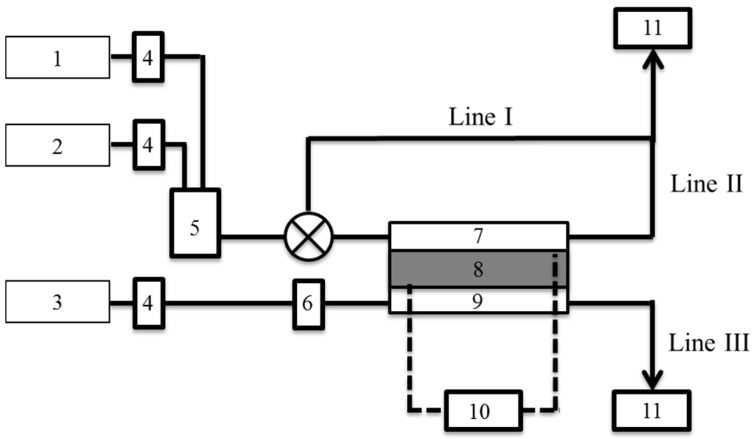
Schematic illustration of experimental measurement of the deuterium separation factor of PEFC. 1. H_2_ gas; 2. D_2_ gas; 3. O_2_ gas; 4. Mass flow controller; 5. Gas mixture unit; 6. Bubbler; 7. Anode; 8. Electrolyte membrane assembly; 9. Cathode; 10. Variable resistor; 11. Q-mass.

The deuterium separation factor, α, of the PEFC was measured by quadrupole mass spectrometry (QMS) (Qulee-HGM 202, Ulvac Corp., Chigasaki, Japan). Figure 1 shows a schematic of the experimental setup. Humidified O_2_ gas was supplied into the cathode at 40 mL·min^−1^. Mixture gases of H_2_ and D_2_ were used at the anode. The mixing ratio was adjusted by a mass flow controller (MC-200SCCM-D, Alicat Scientific, Tucson, AZ, USA). The composition of the exhaust gas from each of Lines I–III was monitored by QMS. Ion currents of mass numbers (*m*) = 2, 3, 4 were recorded at Lines I–II and those of *m* = 18, 19 at Line III.

## 3. Results and Discussion

**Figure 2:**
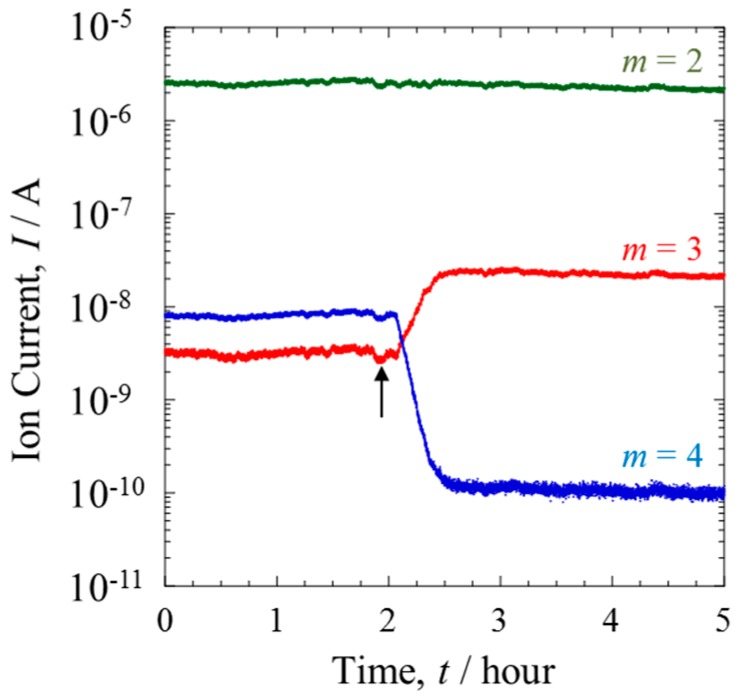
Transient behavior of Q-Mass spectra of mass numbers *m* = 2 (H_2_, green line), 3 (HD, red line) and 4 (D_2_, blue line) at the anode side. A mixture of H_2_ (10.0 mL·min^−1^) and D_2_ (0.1 mL·min^−1^) was passed through the PEFC for 2 h and then passed directly to the Q-Mass for 3 h without power generation.

A mixture gas of H_2_ and D_2_ was supplied to the anode of the PEFC. The effect of the Pt catalyst on the mixture gas was investigated by comparing the gas component composition before and after introducing the anode. With the PEFC switched off, the effect of the catalyst could be studied in the absence of electrochemical kinetic factors arising from power generation by the cell. Figure 2 shows the QMS data of each mass number (*m* = 2–4) when the ratio of D/H was 10^−2^. With the PEFC switched off, the QMS data of Line I indicated no change in the isotopic composition of the gas over time. However, a trace amount of a species with *m* = 3 was detected. This can be attributed to HD, formed via the fragmentation of H_2_ and D_2_ during the ionizing process in the QMS chamber. 

The mixture gas from the PEFC outlet side was monitored from Line II. The arrow in Figure 2 indicates the time when the gas line was switched from Line I to II. The ion current of *m* = 2, corresponding to H_2_, remained almost constant, and was independent of passing through the Pt catalytic layer. In contrast, the ratio of HD to D_2_ was inverted, showing a substantial increase of HD. From the ion currents of each mass number it was calculated that more than 95% of the D_2_ gas was converted to HD. As reported previously [12], the generation of HD gas is proceeded by an isotope exchange reaction, as expressed in Equation (1),

H_2_ + D_2_ → 2HD (1)

The exchange reaction is reported to be enhanced on a Pt surface [16,17]. The high kinetic rate of exchange can be attributed to the well-developed catalyst structure in PEFCs [18]. The catalytic activity is also promoted by the use of nano-sized particles and the uniform distribution of these particles on the supporting materials. Additionally, the gas diffusion layer increases the degree of contact between the mixture gas and the catalyst.

Assuming that the ion currents of each mass number were proportional to the numbers of each molecular species, the effect of the membrane on separation was evaluated. The total amount of D in Line II was about 0.1% less than that of Line I. This loss may have occurred with the penetration of D into Line III or the uptake of D by the Nafion membrane.

The composition of the gas from the cathode was analyzed by the QMS connected to Line III (Figure 1). Pure O_2_ gas was supplied to the PEFC. This gas was fully humidified by protium water before being let in to the PEFC. The bubbler was maintained at 298 K. The two ion currents detected at this line had mass values of *m* = 18 and 19. The species with *m* = 18 was normal molecular water, H_2_O, while the other was H_3_O, produced by the fragmentation of H_2_O. It appears that H_2_O was easily decomposed by ionization in the QMS chamber, probably because of its large molecular size. 

**Figure 3:**
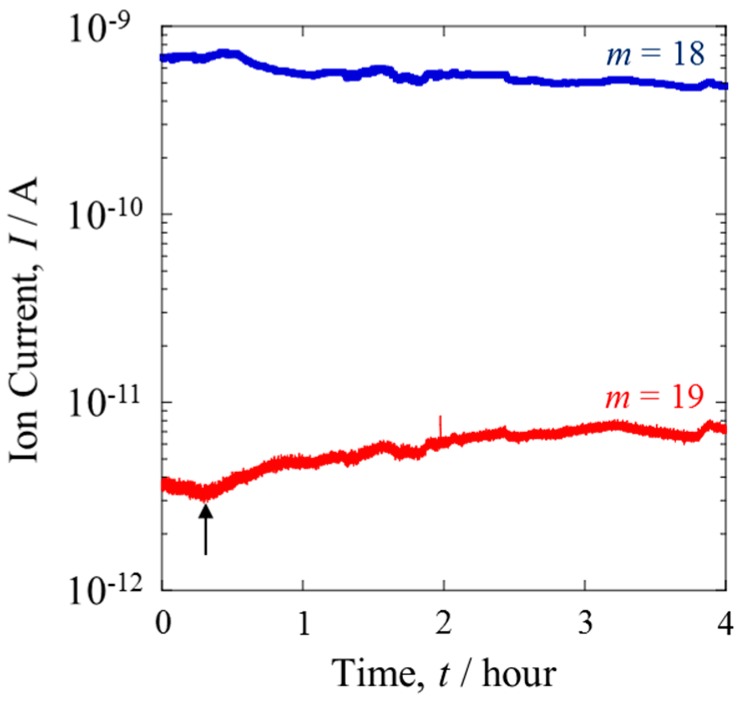
Transient behavior of Q-Mass spectra of mass numbers *m* = 18 (H_2_O, blue line) and 19 (HDO, red line) at the cathode side. Arrow indicates the onset time, when the mixture of H_2_ (10.0 mL·min^−1^) and D_2_ (0.1 mL·min^−1^) was passed to the anode side.

Figure 3 shows the variation of the ion currents of *m* = 18 and 19 when the isotopically mixed gas was supplied to the anode under the same conditions as in Figure 2. The ion current of *m* = 18 decreased over time, while that of *m* = 19 increased such that the ratio of the latter to the former was doubled. Assuming that the frequency of the fragmentation by QMS was independent of the anode condition, this result directly suggests that the increase of the D content resulted from the formation of HDO. The H_2_ and D_2_ species in the mixture gas were oxidized at the anode, resulting in their conversion into H^+^ and D^+^ ions, respectively. The dissociated ions diffused through the conducting polymer toward the cathode. The Pt catalyst facilitated the exchange of the deuterons, D^+^, for H in molecules of H_2_O. This isotope exchange reaction, expressed by Equation (2), was responsible for the increased content of HDO.


D^+^ + H_2_O → H^+^ + HDO (2)

The D/H ratio at Line III was larger than that at the counter-anode. This difference indicates the difference between the diffusion rates of H^+^ and D^+^ in the membrane. 

**Figure 4:**
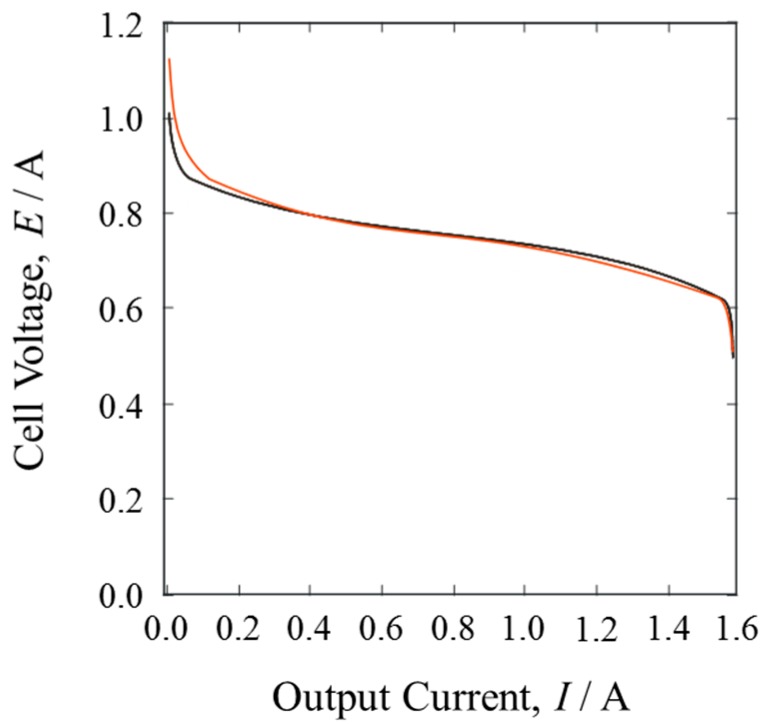
Cell current-voltage curves when PEFC was in operation with pure H_2_ gas (black line) and mixture gases of H_2_ and D_2_ (red line).

The PEFC was connected to the variable resistor and the power performance of the PEFC was measured under current control. Humidified O_2_ gas was used at a flow rate of 40 mL·min^−1^, while dry H_2_ was inlet at 20 mL·min^−1^. Figure 4 shows the current-voltage curves of the pure H_2_ gas and the mixture gases. The open circuit voltage of the pure H_2_ gas was smaller than that of the mixture gases. Since the cathode conditions were the same, the small difference probably corresponded to the isotope effect on the equilibrium potential of HOR [12,19]. 

When the PEFC was in operation, generating electric power, the current-voltage curves of both gases showed almost identical behavior. The cell voltage decreased abruptly at about 1.5 A. In such cells, the cathode potential dominates the cell voltage with an increasing output current, because oxygen reduction on Pt catalysts is inactive under these conditions and gas diffusion is slow. 

**Figure 5:**
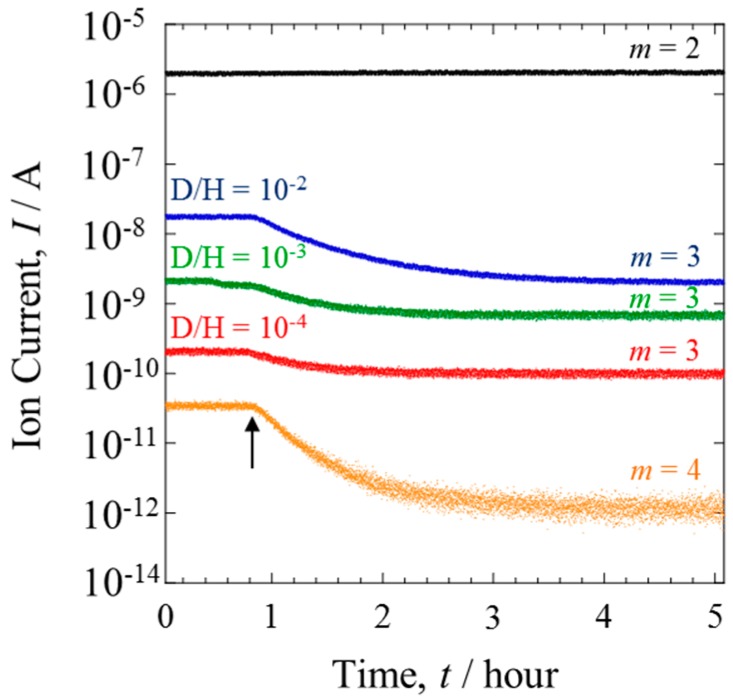
Transient behavior of Q-Mass spectra of *m* = 3 at several D concentrations, D/H = 10^−2^ (blue line), D/H = 10^−3^ (green line) and D/H = 10^−4^ (red line). Arrow indicates the onset time, when the PEFC was switched on. The data of *m* = 2 (black) and *m* = 4 (orange) were measured when the mixture gas with D/H = 10^−2^ was supplied.

The gas composition was monitored in situ. Several inlet H_2_/D_2_ gases with a range of D isotopic concentrations (D/H = 10^−2^–10^−4^) were compared. Figure 5 shows the mass analysis data of the gases from Line II. Before the power generation, the ion current of HD (*m* = 3) changed in accordance with the D concentration of the inlet gas. When the PEFC was operated at 1.2 A, the ion current of HD significantly decreased. The degree of this decrease was lessened with the decreasing D concentration. The contrast with the behavior of H_2_, which showed a constant ion current, is evidence that the D isotope reacted preferentially during HOR [12]. The D selectivity was clearly dependent on the isotope concentration. 

The ion current of D_2_ was detectable only when the mixture gases with D/H = 10^−2^ were investigated. Detection was not possible with lower D concentrations because the current was below the detection limit of QMS. The ion current at D/H = 10^−2^ is also shown in Figure 5. The HOR selectively consumes D_2_ in preference to HD. This is a typical isotope effect, where the variation degree depends on the mass number. 

The deuterium separation factor was calculated by the following equation,

α = ([H]/[D])_a_/([H]/[D])_b_ (3)

where [H] and [D] are the atomic fractions of protium and deuterium, and the subscripts (a) and (b) refer to after and before starting the power generation. The species D_2_ was not considered in the present study because of its low concentration, as shown in Figure 5. The very small ion current of *m* = 4 did not appreciably affect the α values.

**Figure 6:**
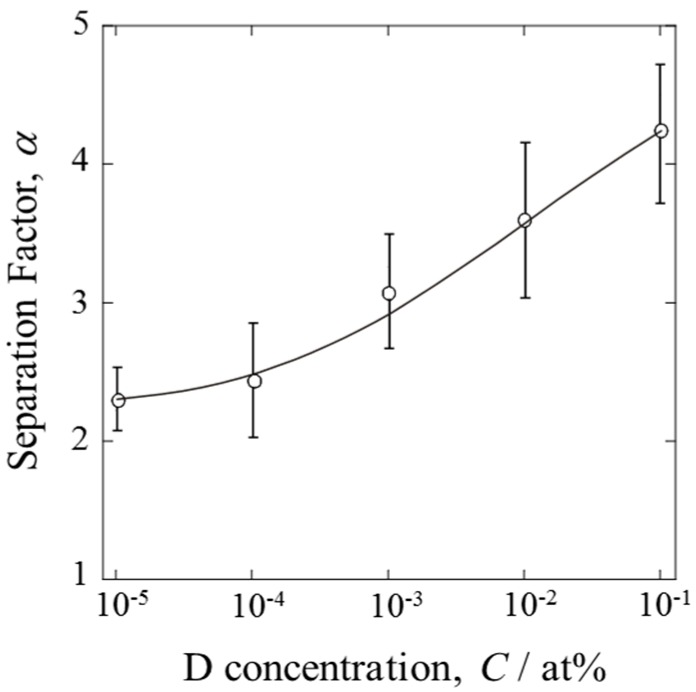
Dependency of separation factor, α, on fuel gas concentration of D when PEFC was operated at 1.2 A.

The separation factors calculated at various D concentrations are shown in Figure 6. The error bars indicate the maximum and minimum values among several experiments. The α values exhibited concentration dependency. D was separated more effectively at higher D concentrations, as expected. However, it should be emphasized that α approached a certain limiting value at low concentrations (D/H < 10^−5^). The PEFC was able to produce water enriched in D. Even dilute mixture gases could be dispersed in the gas diffusion layer, resulting in extensive contact between the gas and the catalyst.

## 4. Conclusions

The D isotopic mass flow in a PEFC with a Nafion membrane was investigated by mass analysis of the mixture gases from both the anode and cathode. Before the power generation was switched on, the mixture gases of H_2_ and D_2_ were almost completely converted to HD at the anode side. A small amount of D could diffuse through the membrane as D^+^ ions and then form HDO at the cathode side by isotopic exchange with protium in H_2_O. The mass balance of D indicated the partial accumulation of D in the membrane.

The power generation of the PEFC was not affected by the introduction of D-containing mixture gases, while the open circuit potential was shifted to a more anodic potential than the equilibrium one of value for isotopically pure H_2_ gas. The D content from the anode side was significantly diluted by HOR. The value of α depended on the D concentration, decreasing from about 4 at D/H = 10^−1^ to about 2 at D/H = 10^−5^.
